# Bridging the gap: Understanding Latino willingness to participate in public health and clinical trials research across diverse subgroups

**DOI:** 10.1016/j.conctc.2025.101440

**Published:** 2025-02-03

**Authors:** Mary A. Garza, Yan Li, Craig S. Fryer, Luciana C. Assini-Meytin, Segen Ghebrendrias, Christina Celis Puga, James Butler lll, Sandra C. Quinn, Stephen B. Thomas

**Affiliations:** a Department of Public Health, College of Health and Human Services, California State University, Fresno, United States; b Central Valley Health Policy Institute, College of Health and Human Services, California State University, Fresno, United States; c Joint Program in Survey Methodology & Department of Epidemiology and Biostatistics, University of Maryland, College Park, United States; d Department of Behavioral and Community Health, School of Public Health, University of Maryland College Park, United States; e Maryland Center for Health Equity, School of Public Health, University of Maryland College Park, United States; f Department of Mental Health, Bloomberg School of Public Health, Johns Hopkins University, Baltimore, United States

**Keywords:** Clinical trial research, Latino population, Willingness to participate in research, Health disparities

## Abstract

**Background:**

The underrepresentation of racial and ethnic minoritized populations in public health and clinical trials research remains a persistent issue. Yet, despite the growing body of literature investigating Latino participation in research, studies examining differences *between* Latino sub-groups remains limited. The purpose of this study was to investigate how knowledge, awareness and willingness to participate in research differs between US- born and immigrant Latinos.

**Methods:**

We conducted a population-based household telephone survey with Latino adults (N = 1264), with 68 % Mexican/Mexican American, 11 % Central/South American, 8 % Puerto Rican and the remaining 13 % self-identified as “Other”. The “Building Trust Survey,” included valid standardized instruments designed to assess knowledge of research, human subjects' protections, previous participation in research, immigrant status (nativity), length of time in the US, and country of origin.

**Results:**

The study found that Latinos who immigrated to the US as teens or young adults were more willing to participate in medical research than those born in the US. Willingness to "take" something in a study varied by Latino subgroup, immigration age, gender, and age. Analysis highlighted that Mexican/Mexican Americans (76 %) and Central/South Americans (74 %) indicated a willingness to participate in research but also were less likely to have been “Asked” to participate in research (9 % and 6 % respectively) compared to the other subgroups (p < .05).

**Conclusions:**

Insights from this study will inform the development of culturally tailored interventions aimed at successfully recruiting and retaining Latino populations in public health and clinical trials research, thereby contributing to more equitable and representative health outcomes.

## 1 Introduction

Minorities remain underrepresented in many public health and clinical trials research studies (herein, research), despite numerous initiatives designed to increase their inclusion [[1], [2], [3], [4]]. This persistent lack of equitable inclusion of certain groups limits the applicability of research results to all Americans, of which Hispanics/Latinos/as/x (herein, Latino) are the largest minority group. According to 2020 US Census reports, the US population of Hispanics is 62.1 million, constituting 18.7 % of the nation's population [5,6]. While participation in research is generally low for all groups [2,4,7], Latinos' participation in research falls short of their representation in the US populations. The NIH reported that 6 % of US-based trials accounted for Latino participants (https://www.ncbi.nlm.nih.gov/pmc/articles/PMC9302767/pdf/nihms-1821755.pdf), a report from the United States Food and Drug Administration (FDA) indicates Latinos make up only 1 % of clinical trials participants [8].

Today, a significant body of literature exists that explores the factors that contribute to Latinos’ willingness to participate, barriers to and incentives for participating, and strategies for improving recruitment and retention of Latino participants. In general, this body of literature suggests that Latinos value the importance of research and are willing to participate in research, including clinical trials [[9], [10], [11], [12], [13]]. A 2014 publication on a survey of 1100 Americans of different racial and ethnic groups indicated that while Latinos reported lower participation in research than other groups, they also indicated they were willing to participate [7,11], suggesting Latinos are willing, but are not aware of how to participate or are waiting to be asked. The articles that report on identified facilitators and barriers to participation list among the main facilitators: the idea of helping others or helping oneself, advancing the state of science, receiving the best treatment available and financial incentives [[14], [15], [16], [17]]. Cited barriers to participation, not listed in any particular order, include concern that insurance will not cover costs associated with participation, fear of being experimented on, lack of time/travel/childcare, vulnerability to deportation, possible wage loss, and Latino underrepresentation in research staff, but where willing to participate and provide consent if prompted by their primary care provider [11,16,18,19].

While many of these facilitators and barriers are largely similar when compared across White, African American, and Latino populations, as the body of research grows, some factors have emerged as significantly greater barriers specifically for Latino participation in research, including clinical trials. These factors include lack of awareness, language barriers, fear related to undocumented legal status, interpretation inaccessibility, and apprehension in accessing medical resources and assistance within a day [11,[20], [21], [22]]. Numerous studies now suggest that Latinos awareness of clinical trials and how to find information about research opportunities lags behind that of Whites, and in some cases behind African Americans as well [20,[23], [24], [25]]. Additionally, some studies have revealed that Latinos value the input of family in the decision making process more than other groups [22,[26], [27], [28]]. Other studies find that Latinos expect their physicians to tell them what to do regarding research participation [22,29] or rely heavily on their doctor's recommendation [26,30], even while only a limited number hear about clinical trials through their providers [11]. However, in their 2014 systematic review, George et al. (2014) reported that evaluated distinct and shared facilitators and barriers to research participation for different racial and ethnic groups reported a “lack of distinct barriers and facilitators for Latinos,” (page e23) which the authors attributed to “the limited articles exclusively focused on Latinos and the greater reporting of their shared experiences with other immigrants,” (page e23) and noted that “less detailed information on Latinos may have resulted from their inclusion in 61 % (11 of 18) of the articles with multiple racial/ethnic groups that may have limited the attention to the nuances distinct to Latinos” (page e23). Thus, there is a lack of research investigating Latino willingness to and interest in participation in research, including clinical trials, and even less information about Latino sub-groups. Marquez, D. X., Perez et al. (2022) reported that low clinical invitations in both English and Spanish, along with unfamiliarity with trials in their native country, contributed to a lack of participation among Latinos.

The emerging paradigm for recruitment in underrepresented populations is to tailor engagement, recruitment and retention efforts to the specific cultural and linguistic characteristics of the target group [31,32]. Our growing knowledge about facilitators and barriers to participation for certain groups helps to define the strategies for recruitment and retention that will be most effective for the population of interest. For Latinos, these strategies might include recruiters who speak Spanish or recruitment materials translated into Spanish, the acceptance that family input into decision making is important and inviting family to be a part of the process [26,27,33] and the use of specially trained community health workers such as *promotoras* [28,[34], [35], [36]].

And yet, despite the growing body of research investigating the reasons Latino participation in and attitudes about research, studies examining specific differences *between* Latino sub-groups remains limited [9,29,37]. The Latino population in the US is very heterogeneous, and little work has been done to investigate how knowledge, awareness and willingness to participate for immigrant Latinos differs from US born Latinos (nativity), how length of time in the US, and country of origin or parental country of origin impacts the willingness to participate or which strategies to engage with and recruit Latino participants will be most effective with certain Latino subgroups. In an effort to address this gap in our knowledge, we analyzed the results of a national survey with 1249 Latino participants to: 1) examine differences in attitudes, knowledge, and willingness to participate in research; 2) understand the barriers and facilitators associated with willingness to participate in research; and 3) examine nativity and age immigrated to the US by willingness to participate in research among different Latino sub-groups; thus contributing new knowledge to the field. This knowledge can be used to help inform specific recruitment and retention strategies based on unique characteristics of the Latino population of interest.

## 2 Methods

Through the work of the University of Maryland, Center for Health Equity, a national telephone survey was conducted by ICF-MACRO from June to December 2010 with 2455 African American and Latino participants. Prospective participants were randomly selected based on telephone exchanges associated with geographic areas of high concentrations of African Americans and Latinos. To identify the appropriate exchanges, directory-listed telephone numbers were mapped and assigned to a specific geographic location (census block group, census tract, or zip code). Stratified sampling was conducted to select telephone numbers with five strata defined by the estimated concentration of African Americans and Latinos within exchanges, that is, exchanges with estimated percentage of households of 1) Hispanic≥60 %, 2) Hispanic = 40–60 %, 3) AA≥60 %, 4) AA = 40–60 %, and 5) Hispanic or AA≥40 %, but not included in strata 1) through 4). The overall response rate was 20.3 %, which is consistent with response rates from other random-digit-dial surveys [38,39]. For the current manuscript, the sample was restricted to Latinos only (N = 1249). The resulting sample represents Latino populations that live in predominantly Latino neighborhoods. University of Pittsburgh Institutional Review Board approved the study and informed consent of all participants was obtained.

### 2.1 Measures

*Socio-Demographics*: Ten socio-demographic variables were measured. There were four Latino subgroups identified: Mexican American, Puerto Rican, Central/South American, and Other. Other demographic variables include gender, age, education, marital status, and income. Education was categorized into two levels: below college and college or above. Marital status was categorized into two levels: married or living with a partner and other. Income was categorized into three levels: 1) below $36,000, 2) $36,000 to $76,000; and 3) above $76,000. Three other variables were health insurance (yes/no), health status, and perceived socio-economic position (SEP). The participants’ current health status was measured on a 5-point Likert scale (1 = poor to 5 = excellent). The perceived SEP question: “think of a ladder with 10 steps as representing where people stand in US, what step would you place yourself on the ladder?”, was measured on a 10-point Likert scale (1 = people who are the worst off to 10 = people who are the best off). Age in US was used as a proxy measure of acculturation. It had 4 levels: a) born in US, b) immigrated to US 12 years old and younger, c) immigrated to US between 12 and 21 years old, and d) immigrated to US 21+ years old.

*Willingness to participate:* Willingness to participate in a future medical research study was measured using a 4-point Likert scale (1 = definitely would not to 4 = definitely would). The variable was dichotomized into yes (probably would, definitely would) or no (probably would not, definitely would not). We then asked about participants’ willingness to participate in specific types of research, which varied in level of risk, using a 4-point Likert scale (1 = very unlikely to 4 = very likely). Three composite scores were computed by averaging the 11 items. There were 5 items on the first factor, “do”. These items were: 1) take a survey, 2) participate in an education program, 3) participate in a group interview, 4) limit or restrict your diet, and 5) do exercises. The second factor, “take”, had 3 items loading on it: 1) take medicine by mouth, 2) take a new drug as part of a test, and 3) receive medication by a needle. Lastly, the third factor, “give”, had 3 items loading on it: 1) give blood, 2) take a DNA test, and 3) give urine.

*Previous participation in research*: Two items assessed previous participation in research: 1) Have you ever been asked to participate in a medical research study? and 2) Have you ever participated in a medical research study. Participants responded yes/no.

*Value of human subjects research*: One item assessed the extent to which respondents viewed medical research with human subjects as positive or negative, using a 5-point Likert scale (1 = very negative to 5 = very positive).

*Motivations for participation*: Respondents were asked “what factors motivate participation in research. “A motivation factor was computed by averaging eleven 3-point Likert scale items (−1 less likely, 0 = no effect, 1 = more likely): a) money, b) curiosity, c) close friend or relatives encouraging, d) close friend or relatives also participate, e) a close friend or relative has or had the disease being studied, f) having the disease that is being studied, g) feeling that the researchers were honest, h) free medical care, i) free transportation, j) the idea of helping others, and k) helping you.

*Patient-provider interaction:* Two items measured interaction with providers: 1) if your doctor wanted you to participate in research, you trust he/she would fully explain it to you; and 2) your doctor would not ask you to participate in medical research if he/she thought it would harm you. Participants responded agree/disagree.

*Beneficiaries of research*: A beneficiaries of research composite score was created by averaging five 4-point Likert scale items (1 = not at all to 4 = a great deal), which asked how much each benefitted from medical research: 1) scientists, 2) your community, 3) your family or friends, 4) you, and 5) the general public.

*Attitudes about research*: Four 5-point Likert scale items measured attitudes about research (1 = never to 5 = always): 1) medical research involves too much risk for the participants, 2) participants in medical research are pressured into participating, 3) patients in medical research get better treatment than other patients, and 4) doctors running medical research care more about the research than about the people they study.

*Researcher Honesty*: One item measured the participants' beliefs regarding researcher honesty: “researchers are always honest with the people they want to participate in their studies” (yes/no).

*Experimentation*: Five items measured the participants’ perceptions of medical research:1) How likely is it that you, or people with the same race or ethnicity as you, might be used as guinea pigs in research studies without your consent? (1 = not likely at all to 3 = very likely); 2) How often, if ever, do you think doctors prescribe medication as a way of experimenting on people without their knowledge or consent? (1 = never to 4 = very often); 3) that doctors have ever given you treatment as part of an experiment without your permission (yes/no); 4) Say that in the past, poor people were used in experiments without their knowledge or consent; and 5) Say that there are laws in place to protect people who are in a research study. For items 4–5, participants responded using a 4-point Likert scale (1 = definitely not true to 4 = definitely true).

*Race matching*: One item asked, “How important would it be to you to have a researcher or research staff who looks like you ask you to participate in a study? Participants responded using a 3-point Likert scale (1 = not important at all to 3 = very important).

### 2.2 Analyses

The analyses were conducted by taking into account the stratified sample design of the survey. We used Taylor linearization variance estimation method when estimating standard errors to account for the differential sample weights and the stratification effect. The sampling weight for each stratum was defined as the number of existing telephone numbers in the stratum divided by the number of selected telephone numbers, subject to the post stratification adjustment using 2009 Census distribution of age, gender, and ethnicity. All of the analyses were conducted by using “survey” package in R (version 3.3.1) software.

The estimated proportion of Latino subgroups by socio-demographic, trust and research participation variables are reported in Table 1, representing Latino adults lived in predominately Latino neighborhoods in the US population. A two-way chi-square tests were performed for testing the independence between Latino subgroups and each categorical variable. ANOVA tests were performed for comparing means across Latino subgroups for each continuous variable.

**Table 1:** Descriptive statistics by Latino sub-groups.

		Mexican – American	Puerto Rican	Central/South American	Other
	Sample size(%)	871 (67.9)	84 (8.3)	116 (10.6)	178 (13.2)
Categorical variables		Sample size(%)			

Gender	Female	535 (46.7)	51 (45)	74 (58.2)	111 (53)
**Age when immigrated to the US∗**	Born in US	593 (65.8)	66 (80.6)	28 (31.3)	94 (46.2)
	≤12 years	85 (10.3)	8 (14.6)	22 (22.2)	31 (22.4)
	12–21 years	76 (13.4)	5 (4.1)	23 (15.8)	12 (11)
	21+ years	108 (10.5)	3 (0.7)	39 (30.6)	39 (20.4)
Education	College or above	369 (43.7)	40 (34.1)	54 (46.2)	96 (55.2)
Marital Status	Married	552 (68.9)	49 (66)	79 (76.9)	107 (71.2)
**Health Insurance∗**	Yes	614 (66.3)	70 (91.3)	78 (63.1)	149 (81.8)
Income	< $36,000	427 (51.9)	42 (64.7)	56 (73.7)	82 (50)
	$36,000 - $76,000	209 (26.8)	17 (19.9)	27 (18.1)	34 (26)
	> $76,000	129 (21.3)	16 (15.4)	16 (8.2)	33 (23.9)
Trust fully explain it to you	Agree	622 (72.4)	65 (77.9)	87 (69)	134 (76.9)
Not ask to participate if harm you	Agree	659 (82.5)	62 (78.2)	80 (72.2)	139 (83.1)
Researcher honesty	Yes	371 (45.3)	29 (38.4)	41 (47.1)	75 (57)
**Have you ever been asked to participate∗**	Yes	113 (8.9)	21 (21.6)	15 (5.4)	42 (32.2)
**How likely used as guinea pigs∗**	Not likely	307 (39.7)	22 (17.4)	47 (51.1)	79 (46.6)
	Somewhat	400 (44.5)	39 (56.3)	47 (41.9)	70 (38.6)
	Very likely	150 (15.7)	22 (26.3)	19 (6.9)	24 (14.7)
Have you ever been treated without your permission	No	711 (82.9)	57 (73.2)	94 (91.8)	154 (90.4)
**Participants in medical research are pressured into participating∗**	Never	174 (24.8)	13 (4.6)	33 (34.9)	37 (20.1)
	Only occasionally	330 (37.5)	30 (37.3)	36 (35.9)	61 (34.7)
	About happen	190 (24.8)	19 (30.1)	26 (21.7)	40 (21.6)
	Most of time	109 (9.9)	12 (25)	11 (6.3)	18 (15.7)
	Always	37 (3)	6 (3)	4 (1.2)	9 (7.8)
Ever participated in research	Yes	75 (68.5)	13 (47.5)	7 (50.6)	30 (67.7)
Willingness to participate	Yes	617 (75.5)	51 (60.1)	77 (74)	104 (65.5)

**Continuous variables**		Mean (Standard Error)			

Age (years)		38.9 (0.75)	44.99 (3.69)	40.61 (2.52)	42.77 (1.89)
Perceived socio-economic position		5.31 (0.11)	5.15 (0.25)	4.86 (0.21)	5.18 (0.32)
Health Status		4.22 (0.06)	4.19 (0.2)	4.47 (0.19)	4.19 (0.15)
Value of research		3.75 (0.06)	3.4 (0.18)	3.72 (0.16)	3.85 (0.15)
Motivations for participation in research		2.3 (0.03)	2.14 (0.11)	2.26 (0.08)	2.37 (0.07)
Beneficiaries of research		3.34 (0.04)	3.18 (0.15)	3.39 (0.11)	3.42 (0.09)
Race matching		1.65 (0.05)	1.79 (0.14)	1.75 (0.08)	1.54 (0.1)
Risk level: do		3.18 (0.04)	3.18 (0.11)	3.21 (0.12)	3.22 (0.08)
**Risk level: take∗**		2.37 (0.05)	2.24 (0.12)	2 (0.12)	2.4 (0.11)
Risk level: give		3.19 (0.05)	2.9 (0.15)	3.16 (0.11)	3.11 (0.07)

(∗p < .05).

Three factors were extracted from the items on willingness to participate in a future study by risk level, using maximum likelihood extraction method with direct oblimin rotation. There were five items loading on the first factor (Cronbach's alpha of 0.78), labeled “Risk Level: Do”. These items were: 1) take a survey, 2) participate in an education program, 3) participate in a group interview, 4) limit or restrict your diet, and 5) do exercises. The second factor, labeled “Risk Level: Take”, consisted of three items with a Cronbach's alpha of 0.81: 1) take medicine by mouth, 2) take a new drug as part of a test, and 3) receive medication by a needle. Lastly, the third factor, labeled “Risk Level: Give”, consisted of three items with a Cronbach's alpha of 0.74: 1) give blood, 2) take a DNA test, and 3) give urine. There were moderate to large correlations among the three factors. The factor scores were computed by averaging the items on each factor.

A logistic regression was performed with outcome variable-willingness to participate in a future medical research. The following predictors were used for the outcome variable: socio-demographic variables, value of human subjects research, previous participation, motivations for participation, patient-provider interactions, beneficiaries of research, researcher honesty, and race matching. The socio-demographic variables were entered in the first step. All other variables were entered in the second step. Several models were tested in order to develop the most parsimonious set of predictors that are driven by theory and results. The interaction between race as categorical variable and each main effect on the prediction of willingness to participate in a future medical research were tested. Archer-Lemeshow (2006) tests were conducted for the goodness of fit of the final logistic regression model with p-value = 0.27, suggesting no evidence of lack of fit of the model.

Finally, a multiple linear regression was performed on each of the three factors extracted from the items on willingness to participate in a future study by risk level as the outcome variables (Do, Take and Give), using the same set of predictors as stated under the logistic regression.

## 3 Results

**Fig. 1:**
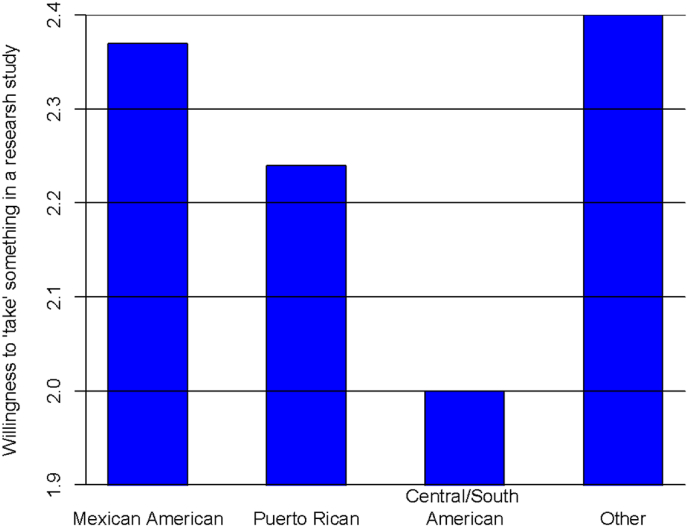
Willingness to “take” something in a research study by Latino sub-groups.

Descriptive statistics for socio-demographic, trust and research participation variables are reported in Table 1 by Latino sub-groups. Nearly 68 % of participants self-identified as Mexican/Mexican- Americans. About 8.3 % of the sample self-identified as Puerto Ricans and about 10.6 % Central/South Americans. The remaining 13.2 % of the sample was categorized as “Other” Latinos (e.g. Cubans, Dominicans, Spaniards). Variables of 1) age when immigrated to US, 2) health insurance, 3) have you ever been asked to participate, 4) how likely to be used as guinea pigs, 5) participants in medical research are pressured into participating, and 6) willingness level of taking something in a research study, were significantly different among Latinos with p-value<0.05. The majority of Mexican/Mexican-Americans (66 %) and Puerto Ricans groups (81 %) were born in US while only 31 % of Central/South Americans were born in the US Larger proportions of Puerto Rican and Other Latinos, compared to Mexican/Mexican American and Central/South Americans Latinos, have health insurance (91 % and 82 % versus 63 % and 66 %) and have ever been asked to participate in research (22 % and 32 % versus 5 % and 9 %). About 25 % of Puerto Rican Latinos think that it is very likely to be used as guinea pigs, and most of times participants are pressured into participating in medical research. In addition, it can be observed that Mexican/Mexican American and Other Latinos have higher willingness to “take” something in a research study than Central/South Americans Latinos. As a consistent observation in Fig. 1, Central/South Americans have a mean value of 2 on willingness to ‘take’ something in a research study while Mexican/Mexican American and Other Latinos have a mean value of about 2.4.

**Fig. 2:**
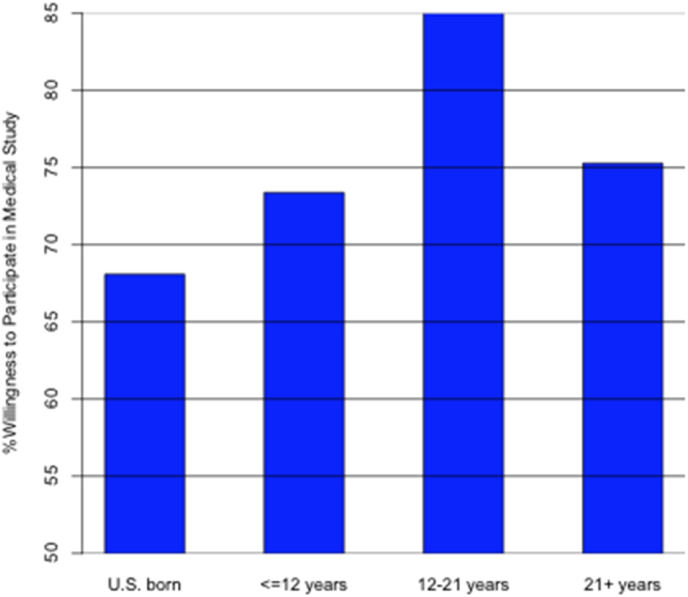
Willingness to participate in a future medical research study by Nativity and Time in US.

Consistent with the observation in Table 1, there was no significant difference in willingness to participate in a future medical study among Latino subgroups in Table 2, after adjusting for other predictors. We found there was a significant difference on willingness to participate in a future medical research study among Age in US with OR = 3.55, similar pattern as shown in Fig. 2 that Latinos who immigrate to US as adolescent/young adults (12–21 years old) were more likely to participate in a medical research study than US born Latinos. We further observed in Table 2 that income level is not associated with the willingness to participate in a medical research study (p-value>0.05). The participants were more likely to participate in a medical research study: 1) as their attitude about the medical research studies were more positive, 2) if more influenced by motivational factors, 3) if their doctors wanted them to participate, and 4) people benefitted from research.

**Table 2:** Adjusted odds ratio and adjusted standardized regression coefficients for Predicting Willingness to Participate and Risk Level of Research.

Variable	Willing to Participate		Risk Level: “Do”		Risk Level: “Take”		Risk Level: “Give”	
	AOR (95 % CI)		β		β		β	
Latinos (ref: Mexican American)								
Puerto Rican	0.34 (0.11, 1.02)		0.096		−0.192		−0.0563	
Central/South American	0.41 (0.16, 1.1)		0.066		−0.444	∗∗	−0.1927	
Other	0.41 (0.15, 1.12)		−0.007		−0.014		−0.2221	∗
Gender (ref: male)	0.85 (0.5, 1.45)		−0.003		−0.198	∗∗	−0.031	
Age	1 (0.98, 1.01)		−0.003		0.012	∗∗∗	0.0032	
Time in US (ref: born in US)								
≤12 years	1.72 (0.51, 5.78)		−0.01		0.083		−0.0618	
12–21 years	3.55 (1.28, 9.86)	∗	−0.002		0.168		0.0564	
21+ years	2.05 (0.77, 5.51)		−0.046		0.333	∗∗	0.1632	
Perceived socio-economic position	1.1 (0.92, 1.32)		0.033	∗	0.021		0.0127	
Education (ref: high school or below)	0.85 (0.5, 1.43)		0.048		−0.144		−0.0221	
Marital Status (ref: other)	0.97 (0.56, 1.7)		0.037		0.066		−0.0814	
Health Insurance (ref: yes)	1.32 (0.72, 2.42)		−0.122		0.011		−0.1286	
Health Status	1.15 (0.92, 1.44)		0.021		−0.047		0.0614	
Income (ref: < $36,000)								
$36,000-$76,000	0.84 (0.45, 1.57)		0.073		−0.03		0.1665	∗
> $76,000	0.99 (0.39, 2.5)		0.141		0.012		0.045	
Value of human subject research	1.83 (1.43, 2.34)	∗∗∗	0.152	∗∗∗	0.178	∗∗∗	0.1463	∗∗∗
Ever been asked to participate in research	1.52 (0.33, 6.94)		0.022		0.229	∗	0.0707	
Motivations for participation in research	5.41 (3.07, 9.52)	∗∗∗	0.287	∗∗∗	0.287	∗∗∗	0.4425	∗∗∗
Patient-Provider Interaction:								
Trust fully explain research to you	2.4 (1.33, 4.32)	∗∗	0.12		0.223	∗	0.1406	
Would not ask if research harms you	0.77 (0.4, 1.45)		−0.206	∗∗∗	0.156		−0.1326	
Beneficiaries of research	2.64 (1.6, 4.34)	∗∗∗	0.205	∗∗∗	0.18	∗∗	0.1767	∗∗
Researcher honesty	1 (0.52, 1.9)		0.083		0.259	∗∗	0.0476	
Race matching	1.44 (0.95, 2.19)		0.074	∗	0.098		0.0231	
Experimentation								
How likely used as guinea pigs	0.72 (0.47, 1.11)		0.051		0.025		−0.1279	∗
Have you ever been treated without your permission (ref: Yes)	0.52 (0.26, 1.06)		−0.043		−0.057		−0.0379	
Participants in medical research are pressured into participating	0.99 (0.78, 1.24)		−0.073	∗∗	−0.079	∗	−0.0644	

(∗p < .05, ∗∗p < .01, ∗∗∗p < .001).

There was no significant difference in willingness to “do” something in a research study among Latino subgroups with p > .05 (Table 2). Participants reporting a higher perceived socio-economic position were more likely to “do” something in a research study than participants reporting a lower SEP (p < .05). The participants were more likely to “do” something in a research study: 1) as their attitude about the medical research studies were more positive, 2) more influenced by motivational factor, 3) if their doctors wanted them to participate, 4) people benefitted from research, 5) researchers look like them, and 6) if they never feel pressure to participating the research.

There was a significant difference in willingness to “take” something in a research study among Latino subgroups with p-value<0.01 (see Table 2). Central/South Americans were less likely to “take” something in a research study than Mexican or Mexican Americans, consistent to the findings in Fig. 1. There was a significant difference in willingness to “take” something in a research study among Time in the US with p-value<0.05. Latinos who immigrated to the US as adults (21+ years old) were more likely to “take” something in a research study than those born in the US. Females were less likely to “take” something than males. Older participants were more likely to “take” something in a research study than younger participants. The participants were more likely to “take” something in a medical research study if: 1) their attitude about the medical research studies were more positive, 2) asked to participate, 3) more influenced by motivational factor, 3) their doctors wanted them to participate, 4) people benefitted from research, 5) they felt medical research study was important, and 6) they never feel pressure to participating the research.

The “Other” Latino group was significantly less likely to “give” something in a research study than Mexican/Mexican American Latinos. Latinos with medium income ($36K∼$76K) are more likely to “give” than those with lower income. There was also no significant difference in willingness to “give” something in a research study among Time in the US, with p-value >0.05. The participants were more likely to “give” something in a research study: 1) as their attitude about the medical research studies were more positive, 2) more influenced by motivational factors, 3) people benefitted from research, and 4) they are not likely to be used as guinea pigs.

## 4 Discussion

This study offers novel insights into the variation in willingness to participate in research among distinct Latino subgroups, as well as their prevailing attitudes toward clinical trials within these communities. It also examines how this willingness varies based on participants' demographic factors, including, but not limited to, nativity and duration of residence in the US Similar to previous studies that explored the range of factors influencing Latino participation in research studies and clinical trials [7,12,13,17], this study reveals that Latinos are generally more willing to participate in a broader spectrum of research studies, including clinical trials than previously recognized. Despite making up nearly 20 % of the US population, according to the 2020 US Census Bureau, Latino participation in public health and clinical trials research remains disproportionately low, even as the population continues to grow, especially among Latino immigrants [40].

To our knowledge, this is the first study that examines research participation among diverse Latino subgroups. The findings of this study challenge existing perceptions, showing that Latinos exhibit a higher willingness to engage in research, including clinical trials, even when studies involve greater potential risks. Specifically, we examined the willingness to participate in research among Mexican-Americans, Puerto Ricans, Central or South Americans, and Other Latinos (e.g., Cubans, Dominicans, Spaniards). Notably, Mexican/Mexican-Americans and Other Latino subgroups were found to demonstrate a greater propensity to participate in research studies that required them to “take” something, compared to Puerto Ricans and Central/South Americans. In the context of this study, “take” was defined as 1) ingesting oral medications, 2) testing new drugs, and 3) receiving injections. Willingness to “take” something in a study varied by Latino subgroup, immigration age, gender, and age. Thus, Latinos are more willing to participate in a wider range of research studies than previously understood, including those with higher degrees of potential risk.

It is documented that Latinos reported lower participation in research than other racial groups; however, they also indicated they were willing to participate [7,11,17]. Our findings confirm Latinos willingness to participate in research. Among Latino subgroups, 76 % of Mexican/Mexican Americans indicated a willingness to participate in research, yet less than 9 % reported ever being asked to participate. Similarly, 74 % of Central/South Americans expressed a willingness to participate, but fewer than 6 % had been asked. In contrast, while 60 % of Cubans were willing to participate in research, 22 % had been asked to do so. Furthermore, positive attitudes, motivation, doctor recommendations, and seeing the benefits of research increased research participation, confirming other studies’ findings [[14], [15], [16], [17]].

Additional findings suggest that both an individual's nativity and time in the US influence their willingness to participate in research. Specifically, Latinos who immigrated to the US as teens or your adults were more likely to participate in research compared to US-born Latinos. These results align with previous studies that have examined the relationship between place of birth, length of stay in the US, and participation in research. For instance, earlier research has proposed that acculturation may contribute to reduced healthcare engagement and research participation among Latinos [41]. Thus, this study's findings suggest that nativity and time spent in the US play a significant role in shaping Latino willingness to participate in research. Continued investigation in this area could help close the gap in Latino participation in research.

The underrepresentation of racial and ethnic minority populations in public health and clinical trials research remains a persistent issue, contributing significantly to health disparities. As highlighted by the NIH (https://www.ncbi.nlm.nih.gov/pmc/articles/PMC9302767/pdf/nihms-1821755.pdf), the recruitment and retention of these populations, including Latinos, poses one of the most critical challenges for researchers. This study sheds light on both the barriers and facilitators influencing the willingness of Latino adults to participate in research, offering key insights that can help address this gap.

The findings underscore the nuanced experiences of different Latino subgroups, particularly the Mexican/Mexican American population. While they were less likely to have a college education, health insurance, or prior experience being asked to participate in research compared to “Other” Latino groups, their higher willingness to participate in future studies is promising. These results point to a clear need for tailored engagement strategies that address the unique socioeconomic and cultural barriers faced by Mexican/Mexican Americans, as well as other Latino groups, in research participation.

Strengths and Limitations: While this study provides valuable insights into the importance of willingness to participate in research, it is not without limitations. Most studies on Latino research participation either include a small Latino sample or group all Latinos together, failing to account for the significant heterogeneity within the Latino population. It is rare for these studies to stratify by Latino subgroups, leading to a lack of nuanced understanding of their unique perspectives. As previously stated, to our knowledge, this study is the first to specifically examine perceptions of research participation among distinct Latino subgroups. Although the data is 14 years old, it remains highly relevant due to the unique and underrepresented nature of the study population. The continued growth and increasing diversity of the Latino population [42], combined with the persistent and widening health disparities they face, underscore the importance of this data. Given the scarcity of stratified research on this issue, the findings provide valuable insights that can inform current and future public health efforts aimed at addressing these gaps. In addition, the overrepresentation of participants from the reference group of Mexican descent may have limited the statistical power to detect differences across smaller sub-groups. We acknowledge this imbalance as a limitation and will explore strategies to achieve better subgroup representation in future research.

Further analysis should enhance our understanding of the role nativity and length of stay in the US play in shaping willingness to engage in research. Additionally, examining the heterogeneity within the “Other” Latino groups will provide a more comprehensive view of the facilitators and barriers influencing research participation. Ultimately, the insights from this study will inform the development of culturally tailored interventions aimed at successfully recruiting and retaining Latino populations in public health and clinical trials research, thereby contributing to more equitable and representative health outcomes.

## CRediT authorship contribution statement

**Mary A. Garza:** Writing – review & editing, Writing – original draft, Methodology, Conceptualization. **Yan Li:** Writing – review & editing, Formal analysis. **Craig S. Fryer:** Writing – review & editing. **Luciana C. Assini-Meytin:** Writing – review & editing, Writing – original draft. **Segen Ghebrendrias:** Writing – review & editing. **Christina Celis Puga:** Writing – review & editing. **James Butler lll:** Writing – review & editing. **Sandra C. Quinn:** Project administration, Methodology, Funding acquisition. **Stephen B. Thomas:** Writing – review & editing, Project administration, Funding acquisition.

## Declaration of competing interest

The authors declare that they have no known competing financial interests or personal relationships that could have appeared to influence the work reported in this paper.
